# P-74. Can Laboratory Investigations Predict Relapsing Malignant Otitis Externa ?

**DOI:** 10.1093/ofid/ofae631.281

**Published:** 2025-01-29

**Authors:** Fatma Hammami, Khaoula Rekik, Fatma Smaoui, Amal Chakroun, Chakib Marrakchi, Makram Koubaa, Mounir Ben Jemaa

**Affiliations:** Infectious Diseases Department, Hedi Chaker University Hospital, University of Sfax, Tunisia, Sfax, Sfax, Tunisia; Infectious Diseases Department, Hedi Chaker University Hospital, University of Sfax, Tunisia, Sfax, Sfax, Tunisia; Infectious Diseases Department, Hedi Chaker University Hospital, University of Sfax, Tunisia, Sfax, Sfax, Tunisia; Infectious Diseases Department, Hedi Chaker University Hospital, University of Sfax, Tunisia, Sfax, Sfax, Tunisia; Infectious Diseases Department, Hedi Chaker University Hospital, University of Sfax, Tunisia, Sfax, Sfax, Tunisia; Infectious Diseases Department, Hedi Chaker University Hospital, University of Sfax, Tunisia, Sfax, Sfax, Tunisia; Infectious Diseases Department, Hedi Chaker University Hospital, University of Sfax, Tunisia, Sfax, Sfax, Tunisia

## Abstract

**Background:**

Malignant otitis externa (MOE) is a life-threatening infection of the external auditory canal. Relapse was reported in 9 to 27% of the cases, mainly due to inadequate length of treatment. We aimed to evaluate the predictor value of laboratory investigations in relapsing MOE.

**Methods:**

We conducted a retrospective study including all patients hospitalized in the infectious diseases department for MOE between 2000 and 2022. Receiver operating characteristic curves and areas under the curves (AUC) were calculated to evaluate the predictor value of laboratory investigations in relapsing MOE.

**Results:**

We included 116 cases, among which 64 were males (55.2%). Relapse was noted in 18 cases (15.5%). The white blood cells count was significantly higher among relapsing MOE cases (10220[8400-10770] /mm^3^ vs 8290[7020-9600] /mm^3^ ; p=0.022). The optimal cut-off of white blood cells count for predicting relapse among MOE cases was 8860/mm^3^, with a sensitivity of 72.2% and a speciﬁcity of 64.2%. The AUC was 0.670 and the 95% conﬁdence interval ranged from 0.544 to 0.796. Neutrophil levels were significantly higher among relapsing MOE cases (6730[5320-7562]/mm^3^ vs 5260[4420-6740]/mm^3^ ; p=0.024). The optimal cut-off of neutrophil level for predicting relapse among MOE cases was 5975/mm^3^, with a sensitivity of 70.6% and a speciﬁcity of 68.4%. The AUC was 0.676 and the 95% conﬁdence interval ranged from 0.545 to 0.806. Creatinine levels were significantly lower among relapsing MOE cases (64[52-91] µmol/L vs 91[70-117] µmol/L ; p=0.013).

**Conclusion:**

White blood cells count and neutrophil levels are a simple blood test that can predict relapsing MOE cases. Such patients require a regular follow-up in order to promptly diagnosis relapse.

**Disclosures:**

**All Authors**: No reported disclosures

